# Insulin-induced changes in skeletal muscle microvascular perfusion are dependent upon perivascular adipose tissue in women

**DOI:** 10.1007/s00125-015-3606-8

**Published:** 2015-05-24

**Authors:** Rick I. Meijer, Erik H. Serné, H. Ibrahim Korkmaz, Donald L. van der Peet, Michiel P. de Boer, Hans W. M. Niessen, Victor W. M. van Hinsbergh, John S. Yudkin, Yvo M. Smulders, Etto C. Eringa

**Affiliations:** Department of Internal Medicine, VU University Medical Center and Institute for Cardiovascular Research, VU University Medical Center, room 4A72, Postbus 7057, 1007 MB Amsterdam, the Netherlands; Department of Pathology and Cardiac Surgery, VU University Medical Center and Institute for Cardiovascular Research, VU University Medical Center, Amsterdam, the Netherlands; Department of Surgery, VU University Medical Center, Amsterdam, the Netherlands; Department of Physiology, VU University Medical Center and Institute for Cardiovascular Research, VU University Medical Center, Amsterdam, the Netherlands; Department of Medicine, University College London, London, UK

**Keywords:** Insulin resistance, Microcirculation, Microvascular recruitment, Perivascular adipose tissue

## Abstract

**Aims/hypothesis:**

Obesity increases the risk of cardiovascular disease and type 2 diabetes, partly through reduced insulin-induced microvascular vasodilation, which causes impairment of glucose delivery and uptake. We studied whether perivascular adipose tissue (PVAT) controls insulin-induced vasodilation in human muscle, and whether altered properties of PVAT relate to reduced insulin-induced vasodilation in obesity.

**Methods:**

Insulin-induced microvascular recruitment was measured using contrast enhanced ultrasound (CEU), before and during a hyperinsulinaemic–euglycaemic clamp in 15 lean and 18 obese healthy women (18–55 years). Surgical skeletal muscle biopsies were taken on a separate day to study perivascular adipocyte size in histological slices, as well as to study ex vivo insulin-induced vasoreactivity in microvessels in the absence and presence of PVAT in the pressure myograph. Statistical mediation of the relation between BMI and microvascular recruitment by PVAT was studied in a mediation model.

**Results:**

Obese women showed impaired insulin-induced microvascular recruitment and lower metabolic insulin sensitivity compared with lean women. Microvascular recruitment was a mediator in the association between obesity and insulin sensitivity. Perivascular adipocyte size, determined in skeletal muscle biopsies, was larger in obese than in lean women, and statistically explained the difference in microvascular recruitment between obese and lean women. PVAT from lean women enhanced insulin-induced vasodilation in isolated skeletal muscle resistance arteries, while PVAT from obese women revealed insulin-induced vasoconstriction.

**Conclusions/interpretation:**

PVAT from lean women enhances insulin-induced vasodilation and microvascular recruitment whereas PVAT from obese women does not. PVAT adipocyte size partly explains the difference in insulin-induced microvascular recruitment between lean and obese women.

**Electronic supplementary material:**

The online version of this article (doi:10.1007/s00125-015-3606-8) contains peer-reviewed but unedited supplementary material, which is available to authorised users.

## Introduction

Arterioles, capillaries and venules make up the microcirculation. Important functions of the microcirculation are to dynamically optimise nutrient and oxygen supply to tissues, and to regulate peripheral resistance [1, 2]. Arterioles regulate flow towards different sites by changing tone [1]. One of the hallmarks of obesity is reduced vasoreactivity, increasing BP and insulin resistance through increased peripheral resistance and decreased delivery of insulin and glucose [3, 4].

Insulin regulates perfusion in the muscle microcirculation, stimulating vasodilation through activation of the phosphoinositide 3-kinase–Akt–endothelial nitric oxide synthase (PI3k–Akt–eNOS) pathway, and concomitantly enhancing vasoconstriction through activating the extracellular signalling regulated kinase 1/2–endothelin 1 (ERK 1/2–ET1) pathway [3]. In insulin-sensitive individuals, activation of the vasodilator pathway dominates, increasing muscle perfusion during hyperinsulinaemia, so-called ‘microvascular recruitment’. In muscle, this augments insulin-stimulated glucose uptake [4–6]. Insulin-induced microvascular recruitment is blunted in insulin-resistant states such as obesity, and in turn contributes to insulin resistance [5, 7, 8]. Why insulin-induced microvascular recruitment is blunted in obesity is unclear.

We recently identified perivascular adipose tissue (PVAT) around skeletal muscle resistance arteries in mice as a new depot of ectopic adipose tissue, and proposed a regulatory role of PVAT in muscle perfusion and insulin sensitivity [9, 10]. In mice, we demonstrated that ex vivo, PVAT exerts paracrine effects on muscle resistance arteries [11]. These paracrine effects enhance insulin-induced vasodilation in lean mice, are mediated by adipokines and are abolished in obesity [11]. The anticontractile effect of healthy PVAT extends previous findings that PVAT controls vascular smooth muscle contractility [11–15]. Changes in PVAT function in obesity may be caused by inflammation [13, 16, 17]. Whether PVAT in the muscle microcirculation enhances insulin-induced vasodilation and microvascular recruitment in insulin-sensitive humans is unknown.

In this study, we hypothesised that PVAT influences insulin-induced vasodilation in the skeletal muscle microcirculation and that its effect differs between lean and obese individuals.

## Methods

### Participants

A total of 15 lean (BMI 18–25 kg/m^2^) and 18 obese (BMI >30 kg/m^2^) female volunteers participated in this study. Because of the different adipose tissue distribution between men and women, only women participated in this study. Physical health was determined by medical history, physical examination and screening blood tests. Inclusion criteria were: female sex, 18–55 years old and white descent. Exclusion criteria consisted of current illness, history of cardiovascular disease, hypertension, diabetes mellitus or impaired kidney function, use of medication affecting endothelial function or glucose metabolism, physical exercise more than three times a week, recent changes in body weight, pregnancy, postmenopausal state, alcohol abuse and smoking. All participants were recruited through advertisements. The study protocol was approved by the medical ethics committee of the VU University Medical Center, and conducted in accordance with the Declaration of Helsinki. All volunteers provided written informed consent before enrolment in the study.

### Study design

Participants visited a quiet, temperature controlled room at the Clinical Research Unit on three separate days within 2 months, the first time for a screening visit, the second time for the hyperinsulinaemic–euglycaemic clamp with contrast enhanced ultrasound (CEU) measurements and the third time for the skeletal muscle biopsy. Participants were fasted overnight for the screening and the clamp, and refrained from physical exercise on the day before the clamp, and before and 2 days after the skeletal muscle biopsy. In three instances, the skeletal muscle biopsy was taken before the clamp—CEU measurements were then performed in the contralateral leg to avoid residual effects of wound healing.

Anthropometry was performed at the screening and before the clamp and fat percentage was assessed by bioelectrical impedance analysis (BF906, Maltron, Rayleigh, UK) [5].

### Hyperinsulinaemic–euglycaemic clamp

**Fig. 1:**
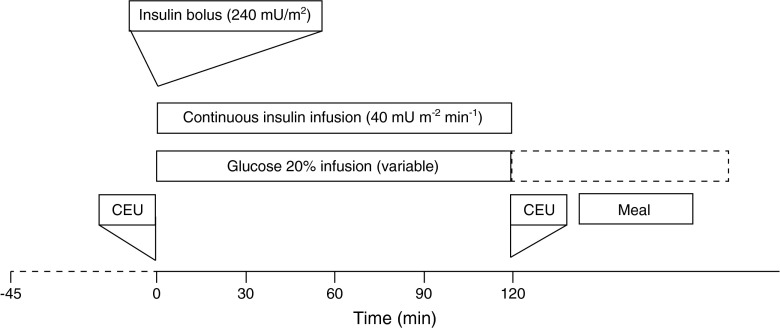
Outline of the hyperinsulinaemic–euglycaemic clamp and microvascular recruitment study day

After arrival at the Clinical Research Unit, the participants acclimatised for 30 min. Insulin sensitivity was determined by a hyperinsulinaemic–euglycaemic clamp, as described previously and depicted in Fig. 1 [18]. Insulin (Actrapid, Novo Nordisk, Bagsvaerd, Denmark) was infused in a primed (240 mU/m^2^), continuous manner, at a rate of 40 mU m^−2^ min^−1^ for 120 min. Euglycaemia was maintained at 5 mmol/l, according to whole-blood venous samples (YSI 2300 STAT Plus Analyzer, Yellow Springs, OH, USA), by adjusting the administration rate of the 20% wt/vol. glucose solution, at 5 min intervals. Whole-body glucose uptake or *M* value, was determined from the glucose infusion rate during the last 60 min of the clamp, and expressed as mg (lean kg)^−1^ min^−1^.

### Microvascular blood volume: CEU

CEU measurements were performed with a Siemens-Acuson Sequoia 512 (Siemens-Acuson, Mountain View, CA, USA), equipped with a 17L5 transducer as described, at the time-points indicated in Fig. 1 [5]. The vastus lateralis muscle was imaged 15 cm proximal of the knee. During the baseline measurement, the probe location was outlined, and landmark structures indicated on-screen. For both the baseline and hyperinsulinaemia measurements, freshly prepared microbubbles (SonoVue; Bracco, Milan, Italy) were infused as an undiluted solution, during constant agitation, at a constant rate of 2.5 ml/min for 4 min in both lean and obese participants. After steady-state microbubble concentration was achieved (2.5 min; electronic supplementary material [ESM] Fig. 1), three real-time inflow curves of 30 s were generated at a mechanical index of 0.28 with linear postprocessing, after destruction of the microbubbles at a mechanical index of 1.7 [5, 19]. Video-intensities (VIs) were analysed using the Image Processing toolbox in MATLAB, version R2011a (Mathworks, Natick, MA, USA). Mean VI during the first 0.5 s was subtracted to correct for large vessels and background noise. Real-time curves from the region of interest in skeletal muscle were fitted to the exponential function VI = MBV[1 − e^−MFV(*t* − 0.5)^], where *t* represents the time (s) after microbubble destruction, MBV is microvascular blood volume, MFV is microvascular flow velocity and e is the natural logarithm (See ESM Fig. 1). CEU does not provide an absolute measure of volume flow (ml min^−1^ [g tissue]^−1^), but is a relative measure used as a paired measurement within participants, and we therefore report only the percentage change in MBV, to minimise the chance that depth or different composition of skeletal muscle (adiposity) affects the outcome [20]. CEU has been shown to correlate with other methods of estimating insulin effects on the microcirculation [5].

### Skeletal muscle biopsy

Surgical skeletal muscle biopsies were taken in the non-fasting state from the vastus lateralis muscle at the same location as the CEU measurement. Local anaesthesia was achieved with lidocaine 2% wt/vol. before the open surgical muscle biopsy of approximately 7 mm × 7 mm × 7 mm. The tissue was immediately stored in ice-cold (0–5°C) MOPS-buffer (in mmol/l: 145 NaCl, 4.7 KCl, 3.3 CaCl_2_, 2.0 MgSO_4_, 1.4 NaH_2_PO_4_, 2.5 pyruvate, 0.02 EDTA, 3.0 MOPS [3-(*N*-morpholino) propanesulfonic acid], 5.6 glucose) and quickly transferred to the laboratory to harvest microvessels for testing.

### Pressure myography

To investigate direct effects of PVAT on insulin-induced vasoreactivity, microvessels were isolated on ice from one half of the skeletal muscle biopsy, and separated from the surrounding PVAT. Microvessels were then mounted on glass cannulae, and randomly assigned to incubation without, or with PVAT. PVAT from alongside its own microvessel was then fastened to one of the cannulae. Ex vivo vasoreactivity of isolated microvessels was studied in the pressure myograph at 80 mmHg and 37°C in K-MOPS (in mmol/l: 125 NaCl, 26 KCl, 3.3 CaCl_2_, 2.0 MgSO_4_, 1.4 NaH_2_PO_4_, 2.5 pyruvate, 0.02 EDTA, 3.0 MOPS, 5.6 glucose and 0.1% wt/vol. BSA) [11]. Microvessels were preconstricted by the potassium in the K-MOPS, and inner diameters recorded to determine baseline diameter. Diameter changes induced by four cumulative concentrations of insulin (0.02, 0.2, 2.0 and 20 nmol/l) were examined for 30 min each. To ascertain having isolated an arteriole or resistance artery, and to check endothelial integrity, acetylcholine (ACh) 1 × 10^−7^ mol/l and ACh 1 × 10^−6^ mol/l was tested at the end of each experiment. At least 10% vasodilation to ACh 1 × 10^−6^ mol/l had to be achieved; otherwise, it was excluded from analysis entirely. Maximum diameter was assessed after administration of papaverine (0.1 mmol/l). Vascular tone was expressed as the percentage of the maximum diameter. The insulin-induced vasoreactivity was expressed as the percentage change from baseline after preconstriction.

### (Immuno-)histology

The second half of the skeletal muscle biopsy was used for histology. This half was stored in buffered formaldehyde (4% wt/vol.) and paraffin-embedded the next morning, 5 μm slices were stained with haematoxylin and eosin. Adipocyte cross-sectional areas were analysed with Image J in a blinded fashion [21]. Only adipocytes at a distance no greater than three adipocytes from the microvessel were included. Macrophage count in PVAT was quantified after CD68 staining and presented as the fraction of the number of adipocytes. For CD68 immunohistochemical analysis, slices were incubated in methanol/H_2_O_2_ (0.3% vol./vol.) to block endogenous peroxidases. Antigens were retrieved by heat inactivation in citrate buffer, followed by incubation with mouse anti-human CD68 (1:400, DakoCytomation, Glostrup, Denmark). Sections were incubated with Envision (undiluted, anti-mouse and anti-rabbit, DakoCytomation). Staining was visualised using 3,3'-diaminobenzidine (DAB 0.1 mg/ml, 0.02% H_2_O_2_ vol./vol.) and counterstained with haematoxylin.

### Statistical analysis

Data were analysed with paired (within group) and unpaired (between groups) *t* tests. Normally distributed data are reported as mean ± SD. Non-normally distributed data were log-transformed, or reported as median and interquartile range and analysed with the Wilcoxon signed-rank test for paired data and the Mann–Whitney test for unpaired data. Pressure myography experiments were analysed using a two-way ANOVA with Bonferroni post hoc test. A *p* value smaller than 0.05 was considered statistically significant. Linear regression analyses were performed to examine the relations between two variables, controlling for age, and standardised *β* coefficients are reported. Bias corrected bootstrapping according to the mediation model by Preacher and Hayes was used to assess mediation effects [22]. In short, multiple regression analyses are performed, and the proposed mediator is important if the effect of the primary factor on the dependent decreases substantially when the proposed mediator is entered. The significance of this mediation is estimated through bootstrapping, where multiple random subsets of the dataset are run to estimate the significance of the change in *β* coefficient; this significance is indicated with a CI. Analyses were performed using IBM SPSS Statistics version 21 (Armonk, NY, USA) and Graphpad Prism 5.01 (La Jolla, CA, USA).

## Results

### Baseline characteristics of the study participants

Baseline characteristics of the participants involved are presented in Table 1. In three women in the lean, and six in the obese group, the skeletal muscle biopsy yielded insufficient tissue for histology, although pressure myography was successful.

**Table 1:** Baseline characteristics

	Lean	Obese	*p* value
*n*	15	18	
Age (years)	42 (25–47)	41 (36–50)	0.34
Weight (kg)	64 ± 7	96 ± 17	<0.001
Height (m)	1.72 ± 0.06	1.70 ± 0.07	0.49
BMI (kg/m^2^)	22.4 (20.1–23.6)	33.0 (31.6–34.3)	<0.001
Waist/hip ratio	0.82 ± 0.07	0.89 ± 0.06	<0.01
Fat percentage	25.0 (20.7–30.4)	44.6 (40.3–46.3)	<0.001
Systolic BP (mmHg)	114 ± 10	127 ± 10	<0.001
Diastolic BP (mmHg)	71 ± 7	74 ± 9	0.36
MAP (mmHg)	85 ± 8	92 ± 8	<0.05
Total cholesterol (mmol/l)	4.9 ± 0.8	4.8 ± 0.9	0.68
LDL-cholesterol (mmol/l)	2.6 ± 0.7	2.7 ± 0.7	0.67
HDL-cholesterol (mmol/l)	1.9 (1.6–2.3)	1.6 (1.4–1.8)	<0.02
Triacylglycerol (mmol/l)	0.8 (0.7–1.1)	1.3 (1.1–1.5)	<0.01
eGFR (MDRD)	87 ± 13	93 ± 20	0.37
Fasting insulin (pmol/l)	30 (25–36)	64 (41–88)	<0.001
Fasting glucose (mmol/l)	4.6 ± 0.4	5.4 ± 1.1	<0.01
HOMA-IR	0.83 (0.78–1.06)	2.12 (1.36–2.80)	<0.001
HbA_1c_ (%)	5.3 ± 0.2	5.6 ± 0.5	0.10
HbA_1c_ (mmol/mol)	35 ± 3	38 ± 6	0.10

Data are means ± SD, or median (interquartile range), depending on the distribution of the data

MAP, mean arterial pressure, MDRD, Modification of Diet in Renal Disease formula (used to estimate the glomerular filtration rate)

### Microvascular recruitment partly explains the difference in metabolic insulin sensitivity between lean and obese women

To assess insulin-induced microvascular recruitment and metabolic insulin sensitivity, CEU was performed before and at the end of the clamp. Metabolic insulin sensitivity (*M* value) was higher in lean (14.7 [12.3–17.6] mg [lean kg]^−1^ min^−1^) compared with the obese women (11.6 [6.0–14.1] mg [lean kg]^−1^ min^−1^), *p* < 0.05. Mean glucose concentration during the clamp was comparable between the lean and obese women, 5.0 ± 0.3 mmol/l vs 4.9 ± 0.3 mmol/l, *p* = 0.29, but plasma insulin was lower in lean (551 ± 103 pmol/l) than in obese women (647 ± 97 pmol/l), *p* < 0.01.

**Fig. 2:**
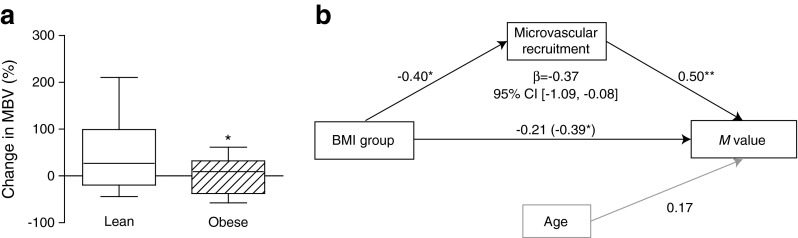
The difference in insulin-induced microvascular recruitment between lean and obese women, and the contribution thereof to metabolic insulin sensitivity. (**a**) Changes in MBV in lean and obese women. Lean women show insulin-induced microvascular recruitment (27% [−19 to +99], *p < 0.05). In the obese women, insulin did not significantly recruit additional MBV (9% [−37 to +32], *p* = 0.97). When these data were log-transformed and tested parametrically (unpaired *t* test), the difference in microvascular recruitment was significantly different between lean and obese women, *p < 0.05. (**b**) Indirect effects of microvascular recruitment on metabolic insulin sensitivity (*M* value). BMI group was related with *M* value with a *β* coefficient of −0.39, *p* < 0.05, corrected for age (horizontal path, between brackets). The *β* coefficient for the BMI group to microvascular recruitment was −0.40, *p* < 0.05 (upsloping path), and the *β* coefficient for microvascular recruitment to *M* value 0.50, *p* < 0.01 (downsloping path). Mediation analysis confirmed the mediating role of microvascular recruitment in the relation between the BMI group and *M* value (*β* = −0.37, CI [−1.09, −0.08]). Indeed, the *β* coefficient from the BMI group to *M* value was attenuated to −0.21, *p* = 0.23 (horizontal path, number outside brackets). *p < 0.05, **p < 0.01. See ESM Table 1 for CIs of the unadjusted and adjusted *β* coefficients from the BMI group to *M* value

During hyperinsulinaemia, MBV increased in lean, but not in obese women (Fig. 2a). Hyperinsulinaemia did not alter MFV in either group. Subsequently we examined whether the change in MBV statistically explains the difference in metabolic insulin sensitivity. Using mediation analyses, microvascular recruitment was indeed identified as a significant mediator in the relation between group (lean or obese) and metabolic insulin sensitivity (Fig. 2b).

### Increased PVAT adipocyte size mediates the relation between obesity and disturbed microvascular recruitment in muscle

**Fig. 3:**
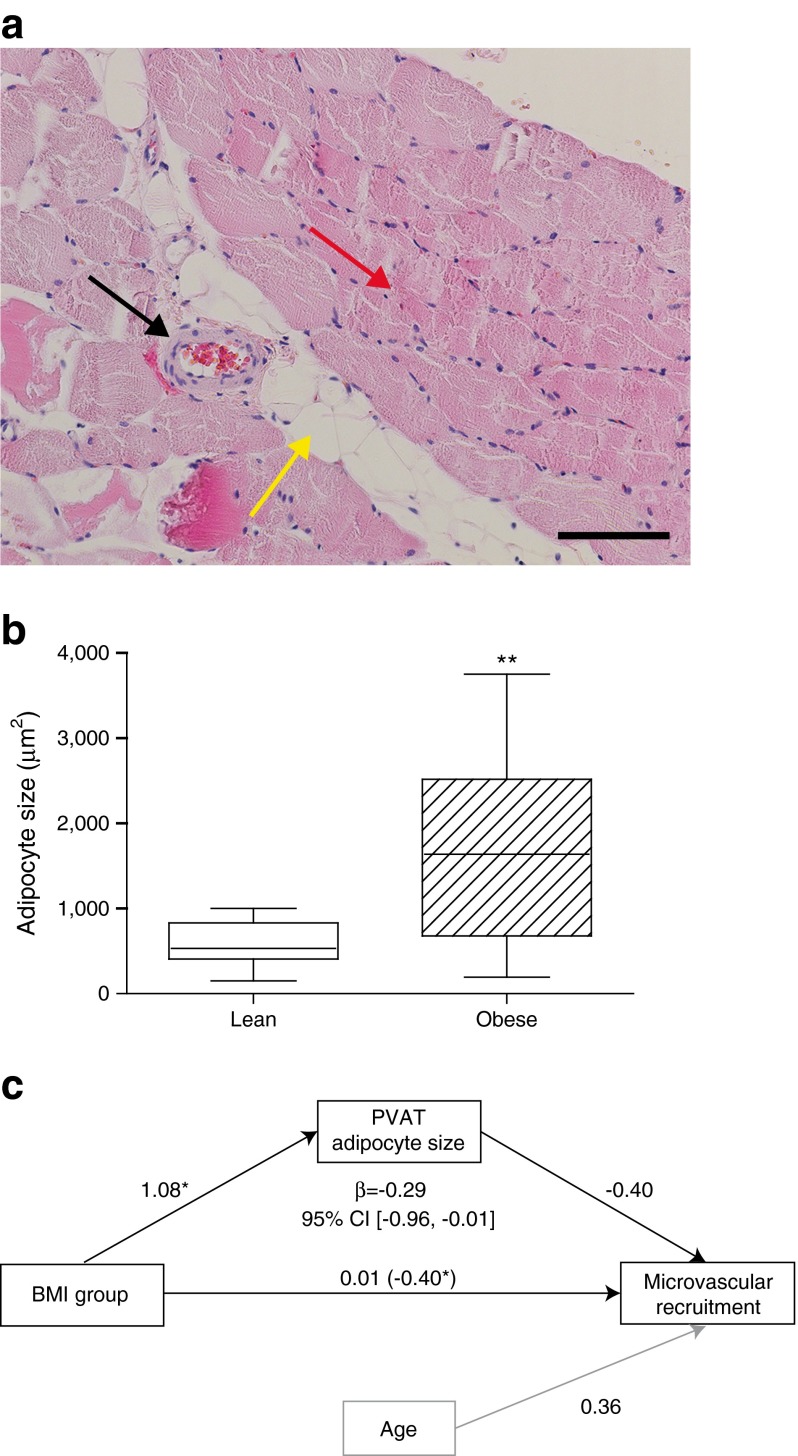
Perivascular adipocyte size partly explains the difference in microvascular recruitment between lean and obese women. (**a**) Typical example of a haematoxylin and eosin staining with an arteriole and microvascular muscle PVAT. Black arrow, resistance artery; yellow arrow, PVAT; red arrow, skeletal muscle. Scale bar ≈ 100 μm. (**b**) In lean women, the size of individual adipocytes in PVAT was smaller than in obese women (530 [407–832] vs 1,637 [679–2,518], *p* < 0.01 [Mann–Whitney *U* test]). (**c**) Indirect effects of perivascular adipocyte size on insulin-induced microvascular recruitment. The BMI group was related to insulin-induced microvascular recruitment with a *β* coefficient of −0.40, *p* < 0.05, corrected for age (horizontal path, between brackets). The *β* coefficient for the BMI group to PVAT adipocyte size was 1.08, *p* < 0.05 (upsloping path) and the *β* coefficient for PVAT adipocyte size to insulin-induced microvascular recruitment −0.40, *p* = 0.065 (downsloping path). Mediation analysis confirmed the mediating role of PVAT adipocyte size in the relation between the BMI group and insulin-induced microvascular recruitment (*β* = −0.29, CI [−0.96 to −0.01]). Indeed, the *β* coefficient from the BMI group to microvascular recruitment was attenuated to 0.01, and no longer significant (horizontal path, number outside brackets), showing that perivascular adipocyte size is a significant mediator in the relationship between the BMI group and microvascular recruitment. **p < 0.01. See ESM Table 2 for CIs of the unadjusted and adjusted *β* coefficients from BMI group to microvascular recruitment

Perivascular adipocyte size was analysed in the biopsies. In obese women, median perivascular adipocyte cross-sectional area was larger than in lean women (Fig. 3a, b), although perivascular adipocyte size did not correlate with BMI within these groups (standardised *B* = 0.259, *p* = 0.42 for the lean, and standardised *B* = −0.367, *p* = 0.22 for the obese).

Macrophage (CD68+) count per number of adipocytes was not different (0.43 macrophage/adipocyte in PVAT of lean women vs 0.25 macrophage/adipocyte in PVAT from obese women, *p* = 0.19). When expressed as macrophages per adipocyte surface area, this was also not different (2.3 [1.7–3.0] in lean vs 1.4 [1.0–3.1] in obese, *p* = 0.36).

We went on to explore whether PVAT adipocyte size in PVAT explained the relation between the study group and microvascular recruitment using mediation analyses. This revealed that PVAT adipocyte size was indeed a significant statistical mediator in the relation between the study group (lean vs obese) and insulin-induced microvascular recruitment (Fig. 3c). Furthermore, perivascular adipocyte size was also related to metabolic insulin sensitivity (*B* = −0.45, *p* < 0.05).

### PVAT from lean women potentiates insulin-induced vasodilation and PVAT from obese women enhances insulin-induced vasoconstriction ex vivo

**Fig. 4:**
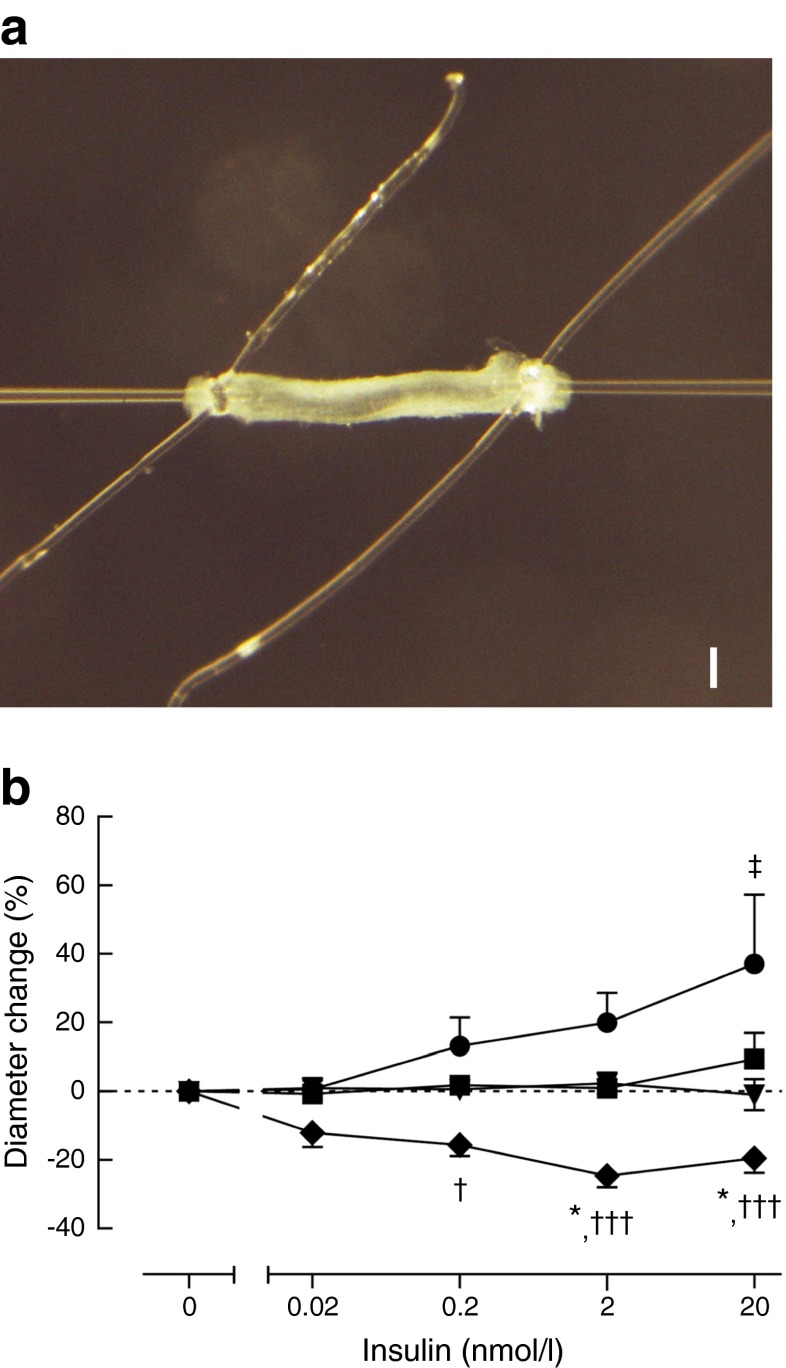
PVAT regulates insulin-induced vasoreactivity ex vivo. (**a**) Example of a cannulated microvessel free from PVAT. Scale bar ≈ 100 μm. (**b**) Without PVAT, microvessels from both lean (triangles, *n* = 6) and obese women (squares, *n* = 8) do not exhibit changes in vascular diameter to increasing doses of insulin in the pressure myograph; but PVAT from lean women (circles, *n* = 8) potentiates insulin-induced vasodilation at the highest concentration, whereas PVAT from obese women (diamonds, *n* = 8) enhances insulin-induced vasoconstriction. *p < 0.05 obese microvessel, no PVAT vs obese microvessel + PVAT; ^†^ *p* < 0.05 obese microvessel + PVAT vs lean microvessel + PVAT; ^†††^ *p* < 0.001 obese microvessel + PVAT vs lean microvessel + PVAT; ^‡^ *p* < 0.05 lean microvessel, no PVAT vs lean microvessel + PVAT

To explore the physiological significance of the statistical relations between PVAT properties and insulin-induced vasoreactivity, and to provide evidence for the causality of the relation, we performed ex vivo pressure myography with microvessels harvested from the skeletal muscle biopsies. Figure 4a shows an example of a mounted microvessel. Failure to dilate 10% to ACh 10^−6^ resulted in exclusion in 50% and 56% of the experiments with lean and obese microvessels, respectively (Table 2).

**Table 2:** Ex vivo microvessel characteristics

	Lean, no PVAT (*n* = 15)	Lean + PVAT (*n* = 15)	Obese, no PVAT (*n* = 18)	Obese + PVAT (*n* = 18)
Number of successful ex vivo experiments of all biopsies performed (%)	6 (40)	9 (60)	8 (44)	8 (44)
Maximum diameter; papaverine, 0.1 mmol/l (μm)	138 (113–167)	164 (103–259)	172 (126–179)	145 (114–193)
ACh mediated dilation (%)	57 (38–70)	48 (43–75)	21 (10–39)*	32 (11–22)
Tone (% of maximum)	20 (12–45)	42 (29–65)	42 (21–76)	29 (18–68)

Data are *n* (%) or medians (interquartile range)

*p < 0.05 compared with lean microvessels without PVAT

Microvessels obtained from lean women and incubated without PVAT showed no insulin-induced changes in diameter ex vivo, comparable with previous murine results [11]. In contrast, microvessels incubated with PVAT from the same individual showed insulin-induced vasodilation (Fig. 4b), supporting the hypothesis that PVAT secretes factors contributing to insulin-induced vasodilation. Microvessels from obese women without PVAT showed no insulin-induced responses in diameter, which did not differ from microvessels of lean women. However, when incubated with their own PVAT, microvessels from obese women constricted in response to increasing doses of insulin (Fig. 4b). These responses were both different from the obese microvessels without PVAT as well as from the lean microvessels with PVAT.

## Discussion

The relation between microvascular PVAT and microvascular vasomotor responses in vivo was hitherto unknown. This study demonstrates a direct relation between PVAT characteristics and insulin’s effects on muscle perfusion. More specifically, perivascular adipocyte size mediates the difference in insulin-induced microvascular recruitment between lean and obese women. These results were extended by ex vivo evidence that PVAT from lean women potentiates the vasodilator effect of insulin, whereas PVAT from obese women causes insulin-induced vasoconstriction. These findings suggest that PVAT regulates insulin-induced vasodilation, and insulin-induced microvascular recruitment in skeletal muscle.

We studied PVAT which abuts the microcirculation, and provide direct evidence for a functional role of PVAT in the regulation of human skeletal muscle perfusion. As others have found, flow-mediated microvascular vasodilation is related to PVAT around the brachial artery [23]. In PVAT and vessels obtained from subcutaneous adipose tissue of lean humans, PVAT shows an anticontractile effect ex vivo in the absence of insulin, which is lost in obesity [12], and can be restored by bariatric surgery [24]. In the latter study, a reduced macrophage count in obese PVAT after bariatric surgery was found. We did not find a difference in PVAT macrophage content between lean and obese women, possibly because our obese participants were less extremely obese and were healthy. A difference in macrophage content in PVAT was also not found by others in high fat diet fed mice, despite altered PVAT function [25]. Macrophage count may therefore not adequately reflect the pro-inflammatory potential of PVAT [13, 26].

Our data demonstrate that insulin-induced microvascular recruitment is a significant mediator in the relationship between obesity and metabolic insulin sensitivity (Fig. 2b). Moreover, our results show PVAT adipocyte size to be a major determinant of the difference in the magnitude of insulin-induced microvascular recruitment between lean and obese women, even though one of the component analyses was of borderline significance (*p* = 0.065; Fig. 3c). These observations suggest that perivascular adipocyte size is more important than being lean or obese per se. Perivascular adipocyte size itself seems an unlikely direct cause of altered PVAT phenotype. More likely, larger adipocyte size is a proxy for altered PVAT characteristics (e.g. hypoxia, inflammation) and therefore relates to an altered secretory adipokine profile [27]. Because we did not find any difference in macrophage infiltration, we did not further explore inflammation as a mediator in the relationship between obesity and microvascular recruitment. Indeed, others have also shown a relationship between adipocyte size and insulin resistance, irrespective of inflammation [28], but also between adipocyte size and adipokine gene expression [29]. Nevertheless, the results show clear mediating effects of microvascular recruitment on metabolic insulin sensitivity, and of PVAT adipocyte size on microvascular recruitment. This means that in order to normalise microvascular responses in obesity, normalising PVAT properties could be of key importance. This is further supported in the study describing PVAT effects after bariatric surgery, where BMI was still in the obese range, but microvascular responses to PVAT were comparable with those of lean healthy participants [24]. Novel ways to decrease adipocyte size, and in particular perivascular adipocyte size, are therefore worth investigating.

The mediation model by Preacher and Hayes was originally designed to study mediation effects in large datasets, but has recently been applied in smaller studies as well. When we studied the same relations by solely looking at the change in *β* coefficient, this yielded similar results, demonstrating the robustness of the data. We decided to report the mediation model results because these provide a more insightful analysis of the mediating effect, together with an estimate of significance.

The results described in Fig. 4 show that PVAT has vasoactive effects in conjunction with insulin. PVAT helps explain the differences in microvascular recruitment between lean and obese participants. As demonstrated, PVAT is necessary for insulin to enhance vasodilation, and therefore insulin-induced microvascular recruitment in vivo. In the absence of PVAT, the microvessels of lean and obese women respond to insulin identically, i.e. they do not change diameter. This suggests that, even though insulin signalling might be different in the endothelium of lean and obese women, this is not sufficient to affect insulin-induced vasoreactivity. However, in the presence of PVAT, a different behaviour of the microvessels is revealed with insulin-induced vasodilation in lean participants, and insulin-induced vasoconstriction in the obese. The divergent responses in the presence of PVAT also demonstrate the dual activation of vasoactive signalling cascades by insulin [30–32]. The importance of PVAT is also demonstrated by previous studies that may not always have removed PVAT properly, thereby potentially influencing their results [33]. Our present results support the hypothesis that PVAT is a functional determinant of microvascular recruitment in skeletal muscle, and therefore of insulin resistance.

Mechanistically, hypoxia and inflammation can alter the adipokines secreted by PVAT. The effects of the hypoxia in obese PVAT can be inhibited with free radical scavengers, improving the effect on microvascular vasodilation [12]. Hypoxia may induce c-Jun N-terminal kinase (JNK) activation in PVAT of obese individuals [11, 34], inhibiting the vasodilator effect of PVAT. On the other hand, adiponectin R1 agonists such as adiponectin have been shown to propagate the vasodilator effects of lean PVAT through signalling via AMP-activated protein kinase α2 (AMPKα2) [11, 12, 35], and its secretion decreases when fat is inflamed. Others have described communication pathways from the endothelium to PVAT, where PVAT function changes in response to endothelial stress in obesity, in order to negate this stress [36, 37]. Our current results cannot confirm or refute that hypothesis, but they at least show that if such a response occurred here, it is incomplete and fails to normalise the microvascular response to insulin.

It is worth mentioning some limitations to this study that need to be considered in conjunction with the results. To the best of our knowledge, this is the first study in which isolated human skeletal muscle microvessels were directly examined in an ex vivo vascular function experiment. Preconstriction was established through 25 mmol/l potassium, which is high compared with interstitial concentrations in vivo, but low compared with other studies examining ex vivo vasoreactivity [12, 24]. PVAT was physically separated from all microvessels to prevent concerns about damaging microvessels during surgery, or mechanical restrictions of PVAT on vasoreactivity. Despite our best efforts, there may be some degree of selection bias of the microvessels inherent in these experiments, possibly favouring larger microvessels. Although diameters did not differ between the two groups, higher orders of microvessels may have been selected in participants with inward remodelling. Different orders of microvessels might be regulated differently during microvascular recruitment, although no evidence exists for that with regard to insulin-induced microvascular recruitment [38]. Moreover, microvascular recruitment measured by CEU is impervious to this theoretical bias, leading us to deem this theoretical bias negligible in this study. Furthermore, as most obese participants had long-standing obesity, they might exhibit long-standing endothelial dysfunction, so failing the quality 10% vasodilation to ACh 10^−6^ may be due to experimental circumstances, or established endothelial dysfunction.

The insulin resistance in the obese group was not extreme, probably due to the exclusion of women with diabetes and hypertension. But despite that, they performed worse with regards to insulin-induced microvascular recruitment and perivascular adipocyte size, compared with our lean women. This shows that even in the phase before the development of obesity-associated complications, PVAT is an important factor, which assumingly would only become stronger were obese women with obesity-associated complications to be included. Despite these reservations, the results align with our own and others’ previous results [11, 12, 15].

Summarising, we have found that PVAT adipocyte size partly explains the relationship between obesity and blunted insulin-induced microvascular recruitment through direct regulation of insulin’s microvascular effects. Therefore, PVAT may be an important target for the treatment of obesity-associated microvascular dysfunction.

## Electronic supplementary material

ESM Fig. 1 (PDF 569 kb)

ESM Table 1 (PDF 9 kb)

ESM Table 2 (PDF 9 kb)

## Abbreviations

ACh: Acetylcholine
CEU: Contrast enhanced ultrasound
MBV: Microvascular blood volume
PVAT: Perivascular adipose tissue
VI: Video intensity
