# Supplementary material for: Insulin-induced changes in skeletal muscle microvascular perfusion are dependent upon perivascular adipose tissue in women
Source: Diabetologia. 2015 May 24;58(8):1907–15. doi: 10.1007/s00125-015-3606-8 (PMC4499111; doi:10.1007/s00125-015-3606-8)
Supplement: Supplementary file 1 — (PDF 569 kb) [file 125_2015_3606_MOESM1_ESM.pdf]

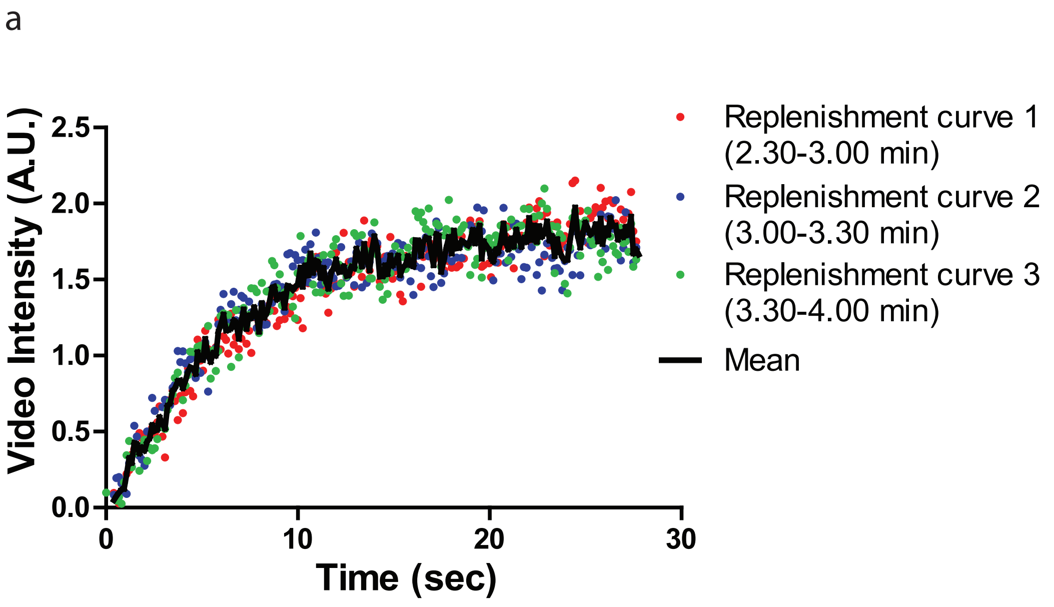

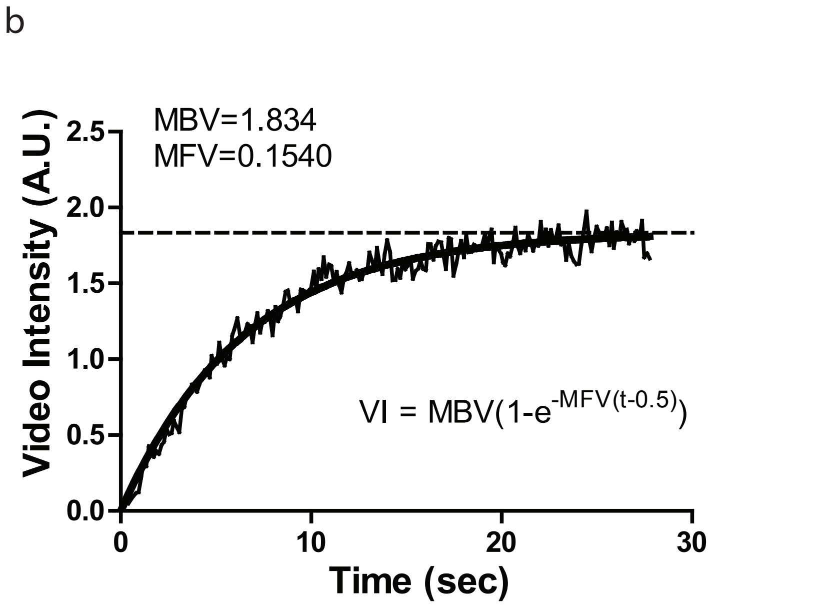

A. Example of three consecutive replenishment curves obtained with CEU to quantify the relative microvascular blood volume (MBV). The black line is the mean of the replenishment curves. The great overlap of the three curves indicates steady-state of the microbubbles was achieved.

B. Curve fit on the mean replenishment curves as shown in (A) according to the formula $VI = MBV(1-e^{-MFV(t-0.5)})$
