# Supplementary material for: Insulin-induced changes in skeletal muscle microvascular perfusion are dependent upon perivascular adipose tissue in women
Source: Diabetologia. 2015 May 24;58(8):1907–15. doi: 10.1007/s00125-015-3606-8 (PMC4499111; doi:10.1007/s00125-015-3606-8)
Supplement: Supplementary file 2 — (PDF 9 kb) [file 125_2015_3606_MOESM2_ESM.pdf]

ESM Table 1

Addendum to Fig. 2b

	Estimate of standardised beta	95% confidence interval
c-path<br>(BMI-group – M-value)	-0.39	[-0.74; -0.05]
c’-path<br>(BMI-group – M-value adjusted for mediation)	-0.21	[-0.56; 0.13]

The betas with confidence intervals of the relation between BMI-group and M-value, unadjusted (c-path) and adjusted (c’-path) for the mediation by microvascular recruitment
